# Sexual dysfunction among youth: an overlooked sexual health concern

**DOI:** 10.1186/s12889-016-3835-x

**Published:** 2016-11-18

**Authors:** Caroline Moreau, Anna E Kågesten, Robert Wm Blum

**Affiliations:** 1 Department of Population, Family and Reproductive Health, Johns Hopkins Bloomberg School of Public Health, 615 North Wolfe Street, Baltimore, MD 21205 USA; 2 Gender, Sexual and Reproductive Health, CESP Centre for research in Epidemiology and Population Health, U1018, Inserm, F-94807, Kremlin Bicêtre, INED, F-75020 Paris, France

**Keywords:** Sexual dysfunction, Sexual health, Youth, Adolescents, France

## Abstract

**Background:**

There is growing recognition that youth sexual health entails a broad range of physical, emotional and psychosocial responses to sexual interactions, yet little is known about sexual dysfunctions and well being in youth populations. This study explored sexual dysfunctions among youth and its associations with other domains of sexual health. Sexual dysfunctions were defined as: problems related to orgasm, pain during intercourse, lack of sexual desire or sexual pleasure.

**Methods:**

Data were drawn from the 2010 French national sexual and reproductive health survey comprising a random sample of 2309 respondents aged 15-24 years. The current analysis included 842 females and 642 males who had sexual intercourse in the last 12 months. Chi square tests were used to test for differences in sexual dysfunctions by sex and explore associations with other domains of sexual health.

**Results:**

Half of females (48%) reported at least one sexual dysfunction versus 23% of males. However, over half (57%) of youth reporting at least one dysfunction did not consider this to hinder their sexuality. Altogether, 31% of females cited at least one sexual dysfunction hindering their sexuality—more than three times the 9% of males. Sexual dysfunction was strongly and inversely related to sexual satisfaction for both males and females and additionally to a recent diagnosis of STI or unintended pregnancy for females. Sexual dysfunctions hindering sexuality were also correlated with a history of unintended pregnancy among males.

**Conclusion:**

While most youth in France enjoy a satisfying sexual life, sexual dysfunction is common, especially among females. Public health programs and clinicians should screen for and address sexual dysfunction, which substantially reduce youth sexual wellbeing.

## Background

The World Health Organization (WHO) defines sexual health as a continuum of physical, psychological, and socio-cultural wellbeing associated with sexuality [1]. Although a growing body of work addresses the complex interrelation of the different domains of sexual health including aspects of sexual wellbeing among adult populations, research on these topics among youth remains scarce. Rather, sexual health research among youth has traditionally taken a risk reduction perspective, mostly concentrating on sexually transmitted infections (STIs) including HIV, unintended pregnancy, and sexual coercion due to their significant contributions to disability adjusted life years for youth [2].

There is growing recognition however, that youth sexual health entails a broader range of physical, emotional and psychosocial responses to sexual interactions than just physical morbidities [3, 4]. Studies in adult populations have revealed high prevalence of *sexual dysfunction* [5, 6], which, according to the International Classification of Disease (ICD-10) [7], encompasses a spectrum of symptoms including lack of sexual desire, lack of sexual pleasure, failure of genital response, orgasmic dysfunction, premature ejaculation and dyspareunia [2, 6]. This symptomatology follows the Masters and Johnson [8] and Kaplan [9] frameworks of the three-phase model of sexual response (desire, arousal, and orgasm), with the addition of sexual pain. Adding to the symptomatology itself, the Diagnostic and Statistical Manual of Mental Disorders (DSM-V) specifies the duration and severity of symptoms to distinguish sexual dysfunction from variation of normal sexual response and the clinical significance of these symptoms [10, 11]. The addition of clinical distress reflects an ongoing debate contrasting a bio-medical model of sexual functioning focusing on physiological response with a psychosocial model that also considers the psychosocial aspects of sexuality including the social expectations of sexual relations [4, 11].

Measuring sexual dysfunction in population-based surveys is challenging given the sensitive nature of the topic, time constraints and potential recall errors [12]. The most widely used instruments are the Female Sexual Function Index (FSFI) (19 items) for females [13] and the International Index of Erectile Function (IIEI) for males [14]. The FSFI instrument only covers a 4-week period, failing to distinguish transient from prolonged symptomatology among females while the IIEI instrument only focuses on erectile dysfunction omitting other functional dimensions of sexual activity among males [6]. None of these instruments assess aspects of sexual distress. While definitions, measures and sampling strategies vary, population based surveys consistently report that the nature and frequency of sexual dysfunctions vary by sex, with females mostly citing hypoactive sexual desire and orgasm while males mostly report premature ejaculation and erectile dysfunction [5, 15].

Furthermore, evidence indicates that many individuals experiencing sexual dysfunctions are not distressed by these symptoms [11]. While sexual dysfunctions emerge early in the sexual trajectories of adults who present with such problems [16], little is known about sexual dysfunctions and their consequences on youth sexual health [15, 17]. The few studies conducted among youth reveal high levels of sexual dysfunction, including pain, lack of desire and failure of genital response [15]. A recent study by Sullivan [18] among 411 Canadian youth aged 16 to 21 years indicated that half of the participants reported at least one sexual functioning complaint. While sexual dysfunctions were frequent, distress related to such dysfunctions was less prevalent: half of those with sexual complaints suffered clinically significant sexual related distress [18].

Sullivan’s study provides a thorough investigation of sexual functioning using validated instruments of male and female sexual functioning [18]. Yet, the small convenience sample of adolescents limits the generalizability of the findings; and the focus on sexual dysfunction and sexual distress alone does not allow an exploration of the interrelation of sexual functioning with other domains of sexual health.

Recent studies conducted in the Great Britain [19] and Flanders, Belgium [20] seek to address some of these gaps, assessing sexual functioning in larger samples of the general population. While the Flanders study reports age-specific prevalence of sexual difficulties and associated distress, the study draws inferences from a convenience sample of the population (online survey advertised through media channels), which raises concern regarding the generalizability of their findings [20]. In contrast, The National Survey of Sexual Attitudes and Lifestyles (Natsal) study in Great Britain assesses sexual functioning among male and female youth, using a nationally representative probability sample [19]. The Natsal survey however uses a different measure of sexual function problems [12] based on a conceptual framework that includes both psycho-physiological and relational aspects of sexual functioning [21]. The Natsal sexual functioning instrument excludes measures of severity and distress, based on the psychometric proprieties of the scale [12]. In addition, questions cover a short period of time (three months) [12].

Building on a more conventional psycho-social and physiological conceptualization of sexual functioning, the present study aims to provide new information on the prevalence of youth sexual dysfunction and its consequences on sexuality in France, and the intersection between sexual dysfunctions with other domains of sexual health, including sexual satisfaction, STIs and unintended pregnancies.

The current study addresses three main questions. What are the patterns of youth sexual dysfunctions and to what extent do young people consider such dysfunctions to affect their sexuality? How do these patterns differ by sex? How are youth sexual dysfunctions related to other domains of sexual health? In this article we refer to sex differences in behaviours and outcomes as we compare males and females without accounting for their gender identity, because gender identity was not assessed in the FECOND study. However, we acknowledge that much of the sex differences that are reported are not only biologically, but also socially driven.

## Methods

### Study design and sample

Data were drawn from the 2010 French national sexual and reproductive health survey, FECOND, comprising 8475 individuals aged 15 to 49 years residing in France. Participants were selected following a two-stage probability sampling method. Phone numbers (including both landline and cell-phones) were generated using random digit dialling. One individual per phone number was selected for participation. The refusal rate was estimated at 20% [22].

After verbal consent, participants responded to a 40-min telephone questionnaire. The FECOND study was approved by the French Commission Nationale de l’Informatique et des Libertés and the current secondary analysis was approved by the Bloomberg School of Public Health Institutional Review Board at Johns Hopkins University.

The present analysis was restricted to respondents aged 15 to 24 years (*n* = 2309) who reported ever having had sexual intercourse (*n* = 944 females and *n* = 731 males). Sexual intercourse was assessed as a positive response to any of two questions “Have you ever had sexual intercourse with a woman?” and “Have you ever had sexual intercourse with a man?” The definition of sexual intercourse did not distinguish between different types of sexual practices. Questions on sexual difficulties and satisfaction were only asked of respondents who reported having had sexual intercourse in the last 12 months (*n* = 886 females and *n* = 679 males). We further excluded participants who stopped responding to the survey before the sexual health module (*n* = 41 females and *n* = 32 males). Our final sample comprised 1484 participants (*n* = 842 females and *n* = 642 males).

### Measures

Topics explored in the multi-thematic FECOND study included socio-demographic status, reproductive histories, past and current sexual health indicators. The key outcome of interest in the present study was *sexual dysfunction* and *sexual dysfunction hindering sexuality* in the past 12 months, assessed through a set of five questions for females and six questions for males. These questions were derived from the last national sexual health survey “The Context of Sexuality in France (CSF)” conducted in France in 2006 for comparative purposes [23]. The CSF sexual dysfunction module was based upon the the ICD-10 classification of sexual dysfunction [7]. The questions examined the following symptoms: lack of sexual desire, lack of pleasure during intercourse, difficulty reaching orgasm and pain during intercourse. In addition, females were asked about vaginal dryness while males were asked about problems of erections and premature ejaculation. Response options assessing the frequency of each sexual difficulty in the last 12 months ranged from “often”, “sometimes”, “rarely”, or “never”. We examined each sexual difficulty separately and constructed a prevalence indicator summarizing the number of problems reported (none, 1, >1). This indicator was based on the four most common sexual problems that were reported among males and females in order to compare results by sex. Following the CSF survey module [23], which not only assessed the frequency of sexual difficulties but how such difficulties related to an individual’s assessment of their own sexuality, respondents were also asked if each of these four components “constituted a problem for their own sexuality”. Based on this information, we constructed a revised set of measures of sexual dysfunctions *hindering* sexuality in the rest of the article.

We further investigated the association between sexual dysfunctions with four other domains of sexual health. First, *history of STI in the last five years* was assessed by a question asking about having had an STI during this time period. If respondents indicated having had an STI in the last five years, they were further asked if the infection were “herpes”, “mycosis” (thrush) or another infectious agent, and in the later case they were asked to provide the name of the infectious agent. Thrush was excluded from the definition of STIs in this analysis. Secondly, *lifetime experience of an unintended pregnancy* was a constructed measure summarizing participant’s pregnancy intentions at the time of each pregnancy. Third, *forced sexual intercourse in the last 12 months* was assessed with a single question asking if the respondent had had forced sexual intercourse against his/her will in the last 12 months (often, sometimes, rarely or never). A dichotomous measure was constructed opposing never to all other responses. Lastly, youth were also asked about *sexual satisfaction* at the time of the survey, operationalized as “very satisfied”, “rather satisfied”, “rather not satisfied” or “not satisfied at all” with current sexual life. We also explored the association between sexual dysfunctions and frequency of intercourse in the last four weeks. All measures were self-reported.

### Statistical analysis

Descriptive statistics were used to explore sex differences in sexual dysfunctions and assess the extent to which each of these dysfunctions hindered sexuality among youth. Using a prevalence indicator of number of reported sexual dysfunctions, we then examined the associations across sexual dysfunctions and other domains of sexual health, including sexual satisfaction, sexual violence, STI, unintended pregnancy and frequency of sexual intercourse. We performed the same analysis assessing associations between sexual dysfunction hindering sexuality and other indicators of sexual health. Chi square tests were used to explore differences in sexual dysfunctions and sexual dysfunctions hindering sexuality by sex and to unveil associations between sexual dysfunctions indicators with other domains of sexual health.

## Results

The mean age of respondents was 20.2 years with no difference by sex (p = 0.23). Most respondents had a partner at the time of the survey, with a greater proportion of females in a cohabitating partnership than males (31% versus 18%) (Table 1). The mean reported age at sexual debut was 16.5 years for females and 15.8 years for males. Males reported a greater number of lifetime sexual partners than females (6.4 versus 3.6, p < 0.001). Frequency of intercourse was equally distributed by sex, with 23% of males and 17% of females reporting no sexual relations in the last 4 weeks. Four percent of respondents reported ever having a same sex partner, with no difference by sex. There were no significant sex differences in the proportion of respondents reporting a history of unintended pregnancy or an STI in the last 5 years. Three percent of respondents reported an experience of forced sex in the last 12 months, with no difference by sex.

**Table 1:** Socio-demographic characteristics and sexual health indicators for sexually experienced youth (*n* = 1484)

	Females *n* = 842	Males *n* = 642	*p*
Age			0.75
15-19 years	45%	46%	
20-24 years	55%	54%	
Relationship status			<0.001
No partner	23%	36%	
Non-cohabitating partner	45%	45%	
Cohabitating partner	31%	18%	
Education level			0.03
< High school graduation	45%	52%	
High school graduation	35%	33%	
> High school	20%	15%	
Professional situation			0.001
Working	28.6%	41%	
Student	58.3%	47%	
Unemployed	10.2%	10%	
Other	2.9%	1%	
Country of birth			0.28
France	92.6%	94%	
Foreign country	7%	6%	
Mean (SD) age at first sexual intercourse	16.5 (16.4-16.7)	15.8 (15.6-16.0)	<0.001
Sexual orientation			0.21
Heterosexual	96%	97%	
Bisexual	3%	1%	
Homosexual	1%	2%	
Mean (SD) number of lifetime partners	3.6 (3.3-3.9)	6.4 (5.7-7.0)	<0.001
Nr of partners last 12 months.			<0.001
1 partner	73%	59%	
2 to 4 partners	23%	33%	
5+ partners	2%	5%	
Missing	1%	2%	
Ever unintended pregnancy	11%	8%	0.18
STI in the last 5 years	5%	2%	0.001
Forced sex last 12 months	3%	4%	0.25
Satisfaction with sexual life			0.02
Very satisfied	59%	51%	
Rather satisfied	34%	42%	
Little satisfied	5%	6%	
Not satisfied at all	2%	2%	
Frequency of sexual acts last 4 weeks			0.11
No acts	17%	23%	
1-4 acts	27%	24%	
5-9 acts	17%	15%	
10+ acts	39%	39%	

### Patterns of youth sexual dysfunctions and sexual dysfunctions hindering sexuality

Female youth were more likely to report sexual dysfunction than their male counterparts (Table 2). Lack of sexual desire and difficulty reaching orgasm were the most commonly cited problems for females: 26% and 31% indicated that these problems occurred on a regular basis (often or sometimes) versus 11% and 8% of males (*p* < 0.001). Pain during sexual intercourse was also more frequent among females: 21% cited that this difficulty occurred often or sometimes versus 4% of males (*p* < 0.001). In addition, 21% of males indicated that they regularly experienced premature ejaculation while a minority (4%) reported problems of erection. One in 11 females (9%) indicated they experienced vaginal dryness on a regular basis.

**Table 2:** Percentage of youth reporting specific sexual difficulties and percentage reporting sexual difficulties hindering their sexuality

	All sexual dysfunctions^a^				Sexual dysfunctions hindering sexuality ^b^			
Type of dysfunction	Frequency	Females *n* = 842	Males *n* = 642	*p*	Type of dysfunction hindering sexuality	Females *n* = 842	Males *n* = 642	*p*
Difficulty reaching orgasm	Often	7%	1%	<0.001	Difficulty reaching orgasm	12%	3%	<0.001
	Sometimes	21%	6%					
	Rarely	19%	10%					
	Never	53%	83%					
Lack of sexual desire	Often	4%	1%	<0.001	Lack of sexual desire	13%	4%	<0.001
	Sometimes	20%	9%					
	Rarely	26%	18%					
	Never	50%	72%					
Pain during intercourse	Often	4%	0%	<0.001	Pain during intercourse	16%	2%	<0.001
	Sometimes	18%	5%					
	Rarely	21%	8%					
	Never	58%	88%					
Lack of pleasure during intercourse	Often	2%	2%	<0.001	Lack of pleasure during intercourse	10%	3%	<0.001
	Sometimes	14%	6%					
	Rarely	26%	18%					
	Never	59%	74%					
Vaginal dryness	Often	1%	-		Vaginal dryness	7%	-	
	Sometimes	8%	-					
	Rarely	9%	-					
	Never	81%	-					
Problem maintaining an erection	Often	-	1%		Problem maintaining an erection	-	2%	
	Sometimes	-	4%					
	Rarely	-	11%					
	Never	-	85%					
Premature ejaculation	Often	-	2%		Premature ejaculation	-	10%	
	Sometimes	-	18%					
	Rarely	-	33%					
	Never	-	48%					
Nr of sexual dysfunctions	None	48%	77%	<0.001	Nr of sexual dysfunctions hindering sexuality	69%	91%	<0.001
	1	29%	17%			18%	7%	
	>1	23%	6%			13%	2%	

^a^ Percentage of youth reporting sexual dysfunctions (often, sometimes, rarely, never) irrespective of whether these problems cause distress

^b^ Percentage of youth reporting sexual dysfunctions hindering sexuality (yes/no)

Using the prevalence indicator of combined sexual dysfunctions common to both sexes, results show that half of females (53%) reported no sexual dysfunctions, while one in five (21%) indicated more than one dysfunction occurring “often” or “sometimes” in the last 12 months. For males, 80% cited no dysfunction while 4% cited more than one dysfunction (*p* < 0.001). The number of dysfunctions reported did not significantly vary by age with 21% of adolescent females 15-19 years and 4% of adolescent males citing more than one dysfunction.

Female youth were more likely to report that a sexual dysfunction affected their own sexuality than male youth. Almost one in three females (31%) cited at least one sexual dysfunction causing a problem for their own sexuality as compared to 9% of males (*p* < 0.001), with no significant differences by age. This sex difference was due primarily to the higher prevalence of sexual dysfunctions (twice as high among females than males) and to a lesser extent to differences in whether these symptoms were perceived to hinder sexuality. Specifically, 59% of all females reporting at least one symptom considered it posed a problem for their own sexuality versus 39% of males (*p* = 0.02). The extent to which dysfunctions caused a problem for one’s sexuality varied by symptom: 44% to 77% of sexual dysfunction symptoms among females and 34% to 52% among males were considered a to cause a problem for one’s sexuality (data not shown). Pain during sexual intercourse was most likely to hinder sexuality for both sexes, followed by problems of erection for males and vaginal dryness for females (Table 2).

### Relationship between sexual dysfunctions and other domains of sexual health

Table 3 presents the associations between sexual dysfunctions or sexual dysfunctions hindering sexuality that were common to both sexes (lack of sexual desire, lack of pleasure during intercourse, difficulty reaching orgasm and pain during intercourse) and other domains of sexual health. Results indicate a strong association between sexual dysfunctions and sexual satisfaction: 74% of females were very satisfied with their current sexual life when they reported no sexual dysfunction versus 36% of those with more than one dysfunction (*p* < 0.001). In the absence of dysfunction, half of males (54%) were very satisfied with their sexual life but that dropped to about one third (29%) when they reported more than one sexual dysfunction (*p* < 0.001). Associations were stronger when respondents reported a sexual dysfunction hindering their own sexuality. Sexual dysfunction alone was not associated with frequency of intercourse, however sexual dysfunction hindering one’s sexuality was related to frequency of intercourse among males, but not among females. Specifically, over half of males (53%) reported having had no sexual intercourse in the last four weeks if they suffered more than one sexual dysfunction hindering their sexuality, more than double that of males who either reported no or one sexual dysfunction hindering their sexuality (*p* = 0.002). While the overall association between sexual dysfunction and a recent STI diagnosis among females was not significant, further analysis indicated that females reporting more than one sexual dysfunction were more likely to report a recent diagnosis of STI as compared to females without such problems (4% versus %, *p* = 0.03). This association was borderline significant in the presence of more than one dysfunction hindering one’s sexuality (4% versus 2%, *P* = 0.07). In addition, sexual dysfunction was related to a history of unintended pregnancy among female youth (*p* = 0.05), and the presence of more than one dysfunction hindering sexuality was borderline related to unintended pregnancy among females (*p* = 0.06). Males who reported more than one sexual dysfunction as opposed to none were more likely to report an unintended pregnancy (19% versus 7% *p* = 0.01); this association was highly significant when considering sexual dysfunction hindering sexuality (37% versus 7%, *p* <0.001). None of the associations were significant for either males or females when examining the relation between sexual dysfunctions and forced sexual intercourse in the last 12 months. Taken together, there were no sex differences in the associations observed.

**Table 3:** Associations between number of sexual dysfunctions^a^ and other domains of sexual health among youth

	Nr of sexual dysfunctions							
	Females *n* = 842				Males *n* = 642			
	None	1	>1	*p*	None	1	>1	*p*
Intercourse frequency last 4 weeks				0.41				0.34
None	18%	15%	18%		22%	20%	38%	
1-4 acts	23%	29%	32%		23%	27%	21%	
5-9 acts	18%	16%	17%		14%	19%	16%	
10 + acts	41%	39%	33%		41%	34%	25%	
Sexual satisfaction				<0.001				<0.001
Very satisfied	74%	53%	36%		54%	44%	29%	
Rather satisfied	22%	40%	51%		39%	51%	46%	
Not very satisfied	3%	5%	8%		5%	3%	18%	
Not at all satisfied	1%	2%	5%		2%	2%	6%	
Forced sex last 12 months	1%	3%	5%	0.27	4%	6%	4%	0.55
STI last 5 years	1%	2%	4%	0.09	1%	0%	2%	0.50
Ever unintended pregnancy	11%	6%	15%	0.05	7%	8%	19%	0.07
	Nr of sexual dysfunctions hindering sexuality							
	Females *n* = 842				Males *n* = 642			
	None	1	>1	*p*	None	1	>1	*p*
Intercourse frequency last 4 weeks				0.82				0.002
None	18%	15%	17%		22%	23%	53%	
1-4 acts	27%	25%	32%		22%	48%	10%	
5-9 acts	18%	18%	15%		15%	11%	16%	
10 + acts	38%	43%	35%		41%	18%	21%	
Sexual satisfaction				<0.001				<0.001
Very satisfied	69%	47%	24%		53%	26%	17%	
Rather satisfied	27%	47%	57%		40%	63%	50%	
Not very satisfied	3%	5%	13%		5%	11%	17%	
Not at all satisfied	1%	1%	7%		2%	0%	16%	
Forced sex last 12 months	2%	4%	4%	0.48	4%	4%	0%	0.81
STI last 5 years	2%	2%	4%	0.25	1%	0%	0%	0.76
Ever unintended pregnancy	10%	9%	16%	0.21	7%	9%	37%	<0.001

^a^ Sexual dysfunctions include: pain during intercourse, lack of sexual desire, problems reaching orgasm, lack of pleasure during intercourse

Table 4 shows the correlations between sex-specific sexual dysfunctions and other sexual health indicators. None of the sex-specific sexual dysfunctions were related to frequency of sexual intercourse, STI, forced sex or unintended pregnancy. Problems of erection and premature ejaculation were both related to sexual satisfaction especially if they were considered to hinder one’s sexuality while there was no significant association between vaginal dryness and sexual satisfaction among females.

**Table 4:** Associations between sex-specific sexual dysfunctions and other dimensions of sexual health among youth

	Females *N*=842			Males *N*=642					
	Vaginal dryness			Problems of erection			Premature ejaculation		
	Yes	No	*p*	Yes	No	*p*	Yes	No	*p*
Intercourse frequency last 4 weeks			0.54			0.33			0.78
No acts	13%	17%		32%	22%		26%	22%	
1-4 acts	32%	26%		29%	23%		25%	23%	
5-9 acts	21%	17%		17%	15%		13%	15%	
10 acts+	35%	39%		22%	40%		36%	40%	
Sexual satisfaction			0.12			0.02			0.09
Very satisfied	45%	61%		39%	51%		41%	53%	
Rather satisfied	43%	33%		40%	42%		50%	40%	
Not very satisfied	6%	5%		12%	5%		8%	5%	
Not at all satisfied	4%	2%		9%	3%		2%	2%	
Forced sex last 12 months	3%	3%	0.69	1%	4%	0.22	4%	4%	0.84
STI in the last 5 years	1%	2%	0.30	3%	1%	0.20	1%	1%	0.94
Ever unintended pregnancy	14%	10%	0.31	7%	8%	0.74	7%	8%	0.63
	Vaginal dryness hindering sexuality			Problems of erection hindering sexuality			Premature ejaculation hindering sexuality		
	Yes	No	*p*	Yes	No	*p*	Yes	No	*p*
Intercourse frequency last 4 weeks			0.79			0.18			0.32
No acts	14%	17%		38%	22%		33%	22%	
1-4 acts	31%	27%		39%	23%		23%	24%	
5-9 acts	21%	17%		0%	15%		13%	15%	
10 acts+	34%	39%		23%	39%		31%	40%	
Sexual satisfaction			0.18			0.02			0.04
Very satisfied	46%	61%		31%	51%		37%	52%	
Rather satisfied	43%	33%		41%	42%		50%	41%	
Not very satisfied	7%	5%		15%	5%		12%	5%	
Not at all satisfied	5%	2%		13%	2%		2%	2%	
Forced sex last 12 months	3%	2%	0.58	3%	4%	0.69	0%	5%	0.15
STI in the last 5 years	0%	2%	0.24	0%	1%	0.72	2%	1%	0.34
Ever unintended pregnancy	11%	11%	0.96	3%	8%	0.25	7%	8%	0.76

## Discussion

While a majority of sexually experienced youth aged 15-24 years in France enjoy a satisfying sexual life (93% of females and 92% of males reported that they were satisfied or very satisfied with their sexual life), this study indicates that sexual dysfunctions are common although for many young people such symptoms are not reported to be a problem for their own sexuality. Specifically, we found that half of females and a third of males reported at least one sexual dysfunction; however, only a third of females and 9% of males reported that the dysfunctions hindered their sexuality.

Our estimates are difficult to compare to existing literature, since such reports are scarce in this age group [17], use different populations or different survey instruments and different time frames to assess the prevalence of sexual dysfunctions and sexual distress [15]. Compared to a recent Flemish study conducted among a convenience samples of 15000 women aged 16 to 74 years recruited online and responding to Sexual Functioning Scale questionnaire, our study showed similar levels of vaginal dryness and absence/delayed orgasm alone among the youth population but lower prevalence rates of lack of desire and dyspareunia [20]. The proportion of sexual problems causing distress was generally above 50% in the Flanders study [20], which is higher than our estimates of sexual dysfunction hindering sexuality. However, sexual distress and the relation of sexual dysfunction to sexuality are two different constructs, the later extending far beyond sexual practice to encompass notions of identity, attitudes and feelings towards sex. In addition, the sexual distress measure of the Flemish study criteria included both personal and partner distress as recommended in the DSM-IV classification, which may have inflated their estimates, as interpersonal distress is no longer a criterion for sexual dysfunction in the DSM-V [10]. Differences in sampling method (convenience sample in the Flemish study versus probability sampling in the FECOND study) may also account for some of the differences observed. The Natsal study in Britain used an extended definition of sexual functioning (incorporating both psycho-physiological and relational aspects of sexual functioning) and a short time frame to assess sexual dysfunction (3 months) [12]. In addition, the Natsal measure did not specifically assess the overall consequences of sexual dysfunctions on the respondent’s own sexuality. While these differences preclude meaningful comparisons with our current study, the Natsal study also reported that a significant proportion of youth had low sexual function (14% of young women and 13% of young men aged 16 to 24 years) [19], calling our attention to address sexual functioning problems across the lifespan.

In our study a substantial proportion of males and females did not consider sexual dysfunctions to be problematic for their own sexuality (41% of females and 61% of males). The gap between symptomatology and related distress is the focus of much debate regarding the diagnostic criteria for sexual dysfunctions [11]. The proponents of a medical model (reflected in the ICD-10 classification) argue that the diagnostic criteria for sexual dysfunction should not include its clinical or psychosocial consequences, while others referring to a socially inspired model of sexual functioning consider distress as an indicator of sexual dysfunction (DSM-V), drawing attention to the functional utility of the definition [11]. This later perspective has gained momentum in the advent of marketing of drugs for erectile dysfunction, bearing on the medicalization of sexual health [4]. While the added value of distress to the specificity of the measure remains controversial, the subjective experiences of sexual functioning should be considered as critical elements underlying healthcare seeking behaviours. The relation of sexual functioning to one’s sexuality extends beyond the notion of sexual distress by considering that sexual functioning can also affect one’s sexual identity and one’s attitudes and feelings related to sexual interactions.

Beyond its prevalence assessments, this study contributes new knowledge in several important ways. First, our results indicate marked sex-differences in the prevalence of sexual dysfunction starting in adolescence, which were not observed in the Natsal survey in Britain [19]. However, similar findings were reported in the previous French sexual health survey (CSF survey) conducted in 2006 in a slightly older population, as women between 18 and 35 years were more likely to report sexual distress than men while the reverse was true after the age of 35 [23].

Expanding on prior work, our results further show that sexual dysfunctions are inter-correlated; 30% of females and 19% of males who reported any dysfunction indicated more than one symptom. These sex differences in the interconnection of sexual functioning problems have been described in other studies among older populations [15] and call attention to the relational context in which sexual interactions occur. The Natsal study stresses the importance of the relational nature of sexual interactions [21], but includes the relational aspect of sexual activity within the sexual functioning scale precluding a direct investigation of the intersection of psycho-physiological and relational attributes of sexual function. Further longitudinal exploration of sexual symptoms and clusters of sexual symptoms, as well as how they affect an individual’s sexuality identity, their attitudes, feeling and relational experiences of sexual activity is warranted to understand how dysfunctions evolve over time and across relationships.

Our third contribution highlights the intersection of sexual dysfunctions with other domains of sexual health. In particular, we found a highly negative correlation between sexual dysfunctions and sexual satisfaction, highlighting the important contribution of sexual function to youth sexual well-being. Importantly, we found that the clustering of dysfunctions was related to a history of STI among females, and increased likelihood of reporting an unintended pregnancy in both sexes. A similar correlation between low sexual function and a past history of STIs was also reported in the Natsal study, although this association was not specific to youth [19].

The current study has a number of limitations. Because of the multi-thematic nature of the FECOND study, we did not use a validated measure of sexual functioning, although our questions were drawn from the national sexual health survey conducted in France in 2006, which captured all dimensions of sexual dysfunctions assessed in the most widely used scales (Female Sexual Functioning Index [13] or the Brief Sexual Function Inventory) [24]. However, unlike validated scales that assess symptoms over a four week period and do not measure the subjective repercussions of sexual dysfunction on sexuality, our construct of sexual dysfunction (difficulties that occur often or sometimes and affect individual’s sexuality over the last 12 months) is more in line with the most recent DSM-V definition of sexual dysfunction, involving symptomatology causing significant distress for a prolonged period the time. Our measurement however, does not include precise estimates of frequency and duration, specified in the DSM-V definition, which requires symptoms to be present between 75 and 100% of the time for a minimum of 6 months [9]. As mentioned above, we also recognize an important difference between sexual distress and dysfunctions causing a problem for an individual’s sexuality.

While we assessed the association between sexual dysfunctions and a number of sexual health indicators, we were not able to examine the association with contraceptive usage, the most proximate determinant of unintended pregnancy, due to the small number of youth with an unmet need for contraception (*n* = 9 females and *n* = 8 males). Likewise, the small percentage of youth engaged in casual sex at last intercourse did not allow for a meaningful exploration of condom use at last sexual intercourse. Small sample sizes also limited the interpretation of results related to forced sexual intercourse.

Because this study was based on cross sectional data, we cannot establish causality. Further research using longitudinal design is needed to ascertain the persistence of sexual dysfunction and sexual dysfunction hindering sexuality over time and their predictive effect on other domains of sexual health. Further investigation is also needed to describe the socio-demographic and contextual factors related to sexual dysfunction and dysfunction hindering sexuality with specific emphasis on partner-related factors given the diversity of relationship experiences in adolescence and early adulthood.

## Conclusion

While most youth in France enjoy a healthy sexual life, sexual dysfunctions are common, especially among females. Public health and clinical programs should screen for and address sexual dysfunction, which substantially reduces youth sexual wellbeing and are related to other common sexual health concerns among youth including STIs and unintended pregnancies.

## Abbreviations

DSM-V: Diagnostic and statistical manual of mental disorders
FECOND: French National Fertility Survey
ICD-10: International statistical classification of diseases and related health problems (10th revision)
Natsal: National survey of sexual attitudes and lifestyles
STI: Sexually Transmitted Infection
WHO: World Health Organization
